# Fiber type composition of the human quadratus plantae muscle: a comparison of the lateral and medial heads

**DOI:** 10.1186/s13047-014-0054-5

**Published:** 2014-12-13

**Authors:** Kristen L Schroeder, Benjamin WC Rosser, Soo Y Kim

**Affiliations:** Current Address: Department of Anatomy and Cell Biology, University of Saskatchewan, 107 Wiggins Rd., Saskatoon, Saskatchewan S7N 5E5 Canada; Current Address: School of Physical Therapy, University of Saskatchewan, 1121 College Drive, Saskatoon, Saskatchewan S7N 0W3 Canada

**Keywords:** Muscle fiber, Myosin heavy chain, Foot, Intrinsic foot muscle, Elderly, Quadratus plantae

## Abstract

**Background:**

The human quadratus plantae muscle has been attributed a variety of functions, however no consensus has been reached on its significance to foot functioning. The architecture of the human quadratus plantae consists of an evolutionarily conserved lateral head, and a medial head thought to be unique to Man. Surveys of human anatomy have demonstrated the absence of either the medial or lateral head in 20% of the population, which may have implications for foot functioning if each muscle head performs a discrete function.

**Methods:**

We investigated the quadratus plantae from eleven formalin-embalmed specimens with a mean age of 84 ± 9 years. Immunohistochemical methods were used to determine the percentage of Type I and Type II muscle fibers in the medial and lateral heads of the quadratus plantae from these specimens.

**Results:**

Results showed striking homogeneity in fiber type composition within an individual, with an average difference in Type I fiber content of 4.1% between lateral and medial heads. Between individuals, however, the ratio of fiber types within the quadratus plantae was highly variable, with Type I fiber percentages ranging from 19.1% to 91.6% in the lateral head, and 20.4% to 97.0% within the medial head.

**Conclusions:**

Our finding of similar fiber type composition of lateral and medial heads within an individual supports the hypothesis that the two heads have a singular function.

## Background

The quadratus plantae is a part of the plantar intrinsic foot muscle compartment, and is involved in stabilizing the foot during activities such as standing and walking [1,2]. A variety of functions have been attributed to the quadratus plantae, ranging from supporting the medial longitudinal arch of the foot [3,4], to assisting plantar flexion of the lesser toes [5,6], and pronation (eversion) of the foot [7]. Despite the wide range of reported functions, no consensus has been reached on the significance of this muscle. Clinically, the quadratus plantae has been implicated in heel pain [8], and may contribute to pathologies that feature weakening of the intrinsic foot muscles, such as in Charcot-Marie-Tooth disease [9].

**Figure 1:**
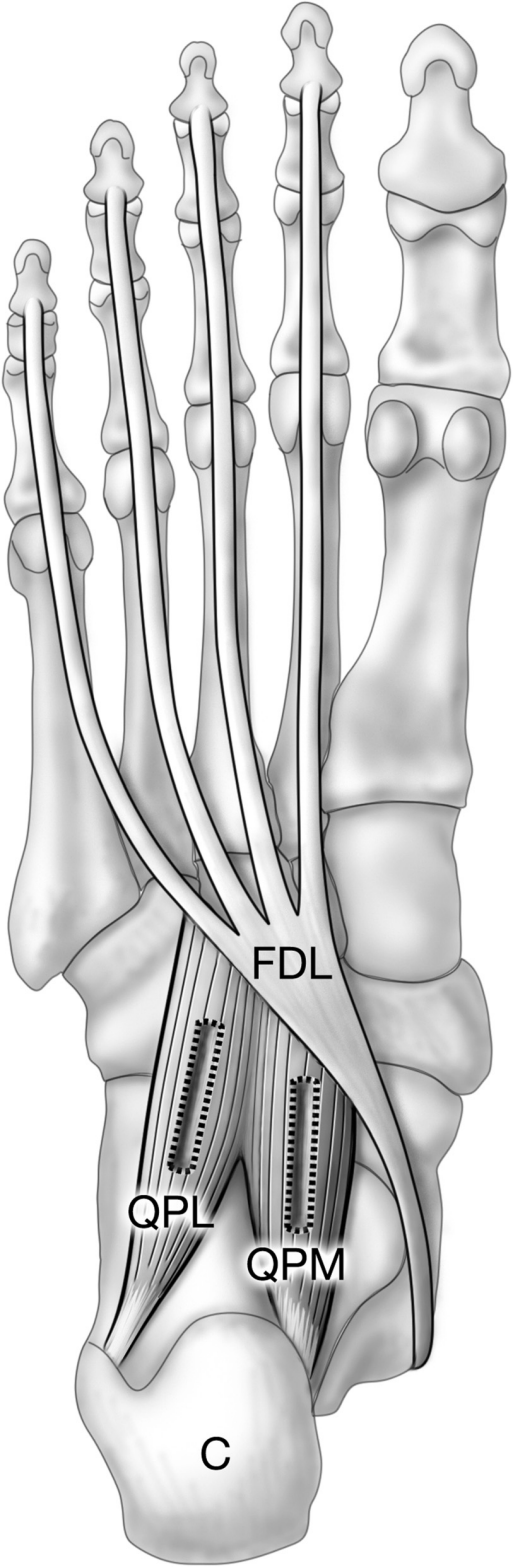
**Location of the quadratus plantae muscle.** Plantar view shows inserting tendon of flexor digitorum longus and quadratus plantae, part of the second layer of intrinsic foot muscles [12]. Quadratus plantae lateral (QPL) and medial (QPM) heads originate from the calcaneus (C) and insert on flexor digitorum longus (FDL). Rectangular boxes indicate the location from which muscle samples were excised.

The human quadratus plantae is formed by two muscle heads; medial and lateral, with the former thought to be unique to Man [10,11]. Most commonly, the evolutionarily conserved lateral head is smaller than the medial head, and originates from the lateral border of the inferior calcaneal surface (Figure 1). The medial head arises from the medial concave surface of the calcaneus and joins the lateral head in a common flat band that inserts into the tendon of flexor digitorum longus. It has been reported that approximately 20% of the human population lacks either the medial or lateral head, though rarely (2%) is the quadratus plantae lacking in its entirety [6,10].

The function of a skeletal muscle is directly correlated to the nature of its constituent fibers [13]. Human muscle fibers are categorized into two principal types based on biochemical and electrophysiological characteristics: Type I and Type II [14,15]. Slow-contracting Type I fibers are resistant to fatigue and depend upon aerobic metabolism to provide energy for contraction. By comparison, fast-contracting Type II fibers fatigue more quickly and have a greater reliance upon anaerobic metabolism. Fiber types are determined by the expression of myosin heavy chain isoforms, with fibers expressing either predominantly type I, type IIA, or type IIX isoforms [16]. A variety of physiological and pathological processes can result in the expression of multiple myosin isoforms within the same fiber, resulting in hybrid muscle fibers that may exhibit altered contractile properties [17,18].

The fiber type content of a muscle varies between regions or compartments that are architecturally and functionally distinct [14,19,20]. Given the various functions proposed for the quadratus plantae, it is possible that the two heads of this muscle have different roles in foot function. A systemic difference in muscle fiber type proportions could indicate a difference in muscle function between the medial and lateral heads, therefore we used immunohistochemical methods to investigate the constituent fiber types of quadratus plantae excised from human cadavers. This study presents the first fiber typing data on the quadratus plantae in humans and provides insight into the composition of this muscle in older adults. Results of our study showed striking homogeneity in the fiber type content of the lateral and medial heads of the quadratus plantae, suggesting a shared function between these two regions.

## Methods

### Cadaveric specimens

Eight female and three male human cadaveric foot specimens were obtained from the Department of Anatomy and Cell Biology, University of Saskatchewan. Each specimen was from a different individual, with four left and seven right feet included in this study. In all cases, embalming in 3.3% formalin occurred less than 24 hours post-mortem following which an interval between 1.5 and 2.5 years elapsed before the quadratus plantae was dissected. Mean age of individuals was 84 ± 9.0 (range 64-96) years. According to available medical information none had any history of neuromuscular disease, and specimens with major foot deformities or apparent joint disease were excluded from our study. Two additional individuals presented with only one muscle head, and were also excluded from this study. Ethics approval was obtained from the Biomedical Research Ethics Board, University of Saskatchewan (permit number 08-197).

### Tissue sampling and cryosectioning

Samples approximately 0.5 × 0.5 × 2-3 cm were excised by blunt and sharp dissection from medial and lateral heads of the quadratus plantae of each specimen (Figure 1). The long axis of each sample ran parallel to the direction of the muscle fascicles. Samples were then coated with Tissue Tek OCT Compound (Sakura Finetek, Torrance, USA), rapidly frozen in 2-methylbutane cooled by liquid nitrogen [15] and stored at -20°C.

Serial cross-sections of each specimen were cut to a thickness of 12 microns in the chamber of a Minitome PLUS Cryostat (Triangle Biomedical Sciences, Durham, USA) at -23°C. Pairs of successive serial sections were picked up on chilled ProbeOn Plus charged microscope slides (Fisher Scientific, Nepean, Canada) and quickly thawed. Consecutive slides were numbered and allowed to air dry for approximately 30 minutes at room temperature followed by storage at -20°C.

### Immunohistochemistry

Immunohistochemical techniques follow our earlier protocols [21,22], and were used to label consecutive slides prepared from lateral and medial quadratus plantae samples. Briefly, slides were removed from the freezer and air dried for 15 minutes. Blocking solution comprised of 2% bovine serum albumin and 5 mM ethylenediaminetetraacetic acid in phosphate buffered saline (0.02 M sodium phosphate buffer, 0.15 M sodium chloride, pH 7.2) was then applied to the slides for 30 minutes. Subsequently, primary monoclonal antibodies were used to label myosin heavy chains of Type I fibers (antibody A4.951) or Type II fibers (antibody A4.74). Primary antibodies utilized in this study were raised in mouse and obtained as hybridoma supernatant from the Developmental Studies Hybridoma Bank (DSHB; University of Iowa, Iowa City, USA). Primary antibody solutions consisted of antibody diluted in blocking solution, A4.951 at a 1:20 dilution and A4.74 at 1:50, and were applied to tissue sections overnight at 4°C. A very low frequency of fiber-like structures not labelled by primary antibody A4.74 or A4.951 were detected, and an antibody directed against the myosin heavy chains of all fiber types (antibody A4.1025, DHSB; used at a 1:20 dilution) was used to confirm these unlabelled structures as muscle fibers. As the ability of antibody A4.74 to recognize the Type IIX myosin isoform has not been definitively resolved [23,24], these unlabelled fibers may represent Type II fibers containing purely IIX myosin.

**Figure 2:**
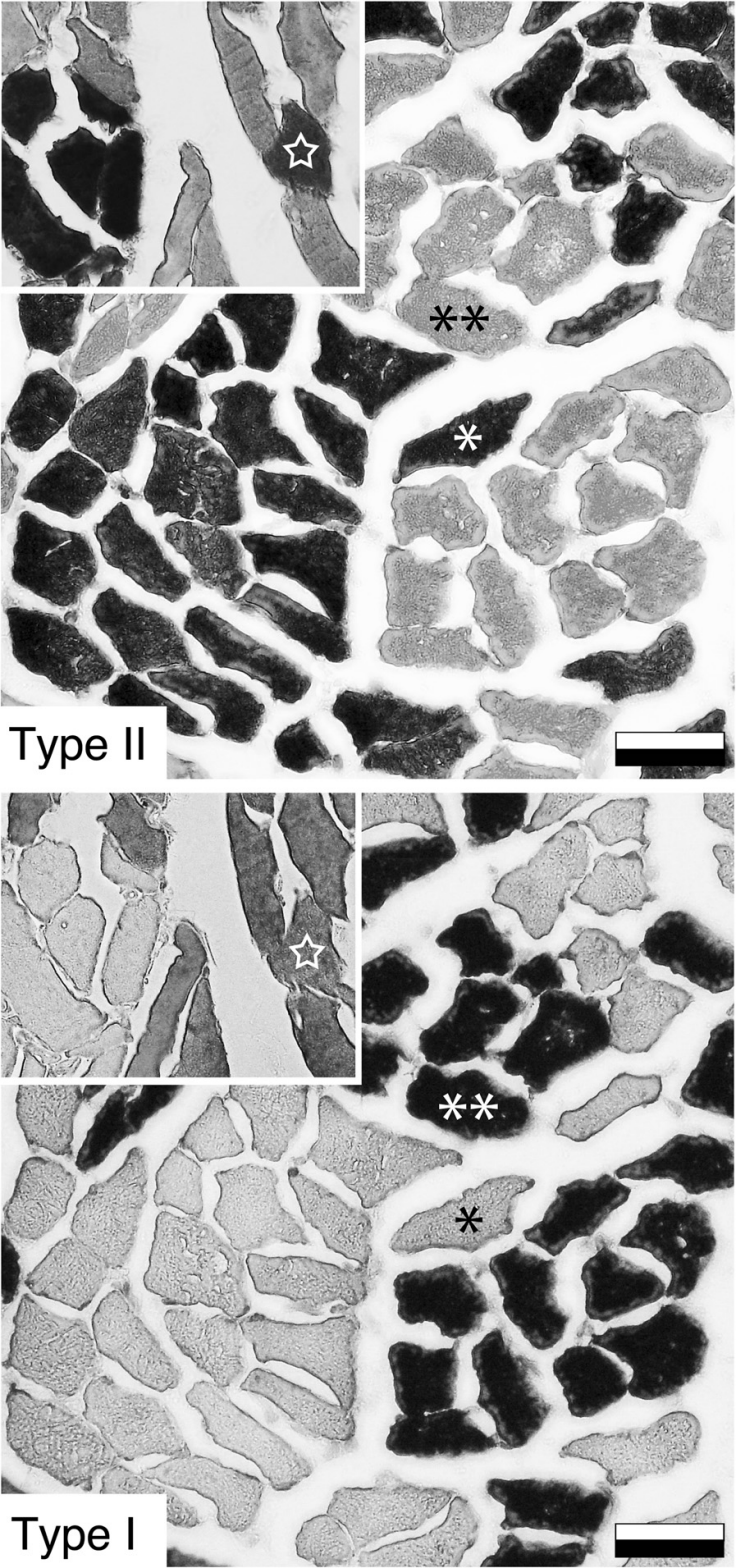
**Immunohistochemical labelling of representative serial cross-sections of the quadratus plantae lateral head. Top:** Muscle fibers labelled for Type II (fast) myosin heavy chains with antibody A4.74 (positive labelling appears as dark staining). **Bottom:** Muscle fibers labelled for Type I (slow) myosin heavy chains with antibody A4.951. Single asterisk indicates a fiber classified as Type II, demonstrated by positive labelling in A and absence of labelling in B. Double asterisk indicates a fiber classified as Type I, demonstrated by positive labelling in B and absence of labelling in A. **Insets:** Cross-section of a different individual exhibiting a hybrid fiber (star), classified based on positive labelling by both antibodies. In both individuals muscle tissue exhibits grouping of fibers into patches of similar types. Scale bar: 50 microns.

The secondary antibody process utilized the Avidin Biotin Complex (ABC) method, and two commercially available kits; the ABC kit (Vector Laboratories, Burlington, Canada; PK-6200) and the DAB kit (Vector Laboratories; SK-4100). As per manufacturer instructions, the included universal secondary biotinylated IgG antibody was used to detect bound primary antibody. A 3% hydrogen peroxide solution in methanol was applied to prevent background staining. An avidin and biotinylated horseradish peroxidase macromolecular complex reagent, also included in the ABC kit, was pipetted onto tissue sections and incubated with tissue sections for 60 minutes in the dark at room temperature. Finally, using the DAB kit, a peroxidase substrate containing 3,3′diaminobenzidine was added to effect a specific colour reaction visible by light microscopy. Slides were then rinsed, mounted using Citifluor (Canemco and Marivac, Canton de Gore, Canada), and stored at 4°C. An example of immunohistochemical labelling is demonstrated in Figure 2.

### Image analysis and fiber type calculations

Images of labelled tissue sections were captured with a Sony Cybershot DSC V3 digital still camera (Sony, Tokyo, Japan) attached to a Zeiss Axioskop 20 microscope (Carl Zeiss, Oberkochen, Germany). Images were then uploaded onto an iMac computer (Apple Computer, Cupertino, USA) for classification and calculation of fiber types.

The antibody labelling evident in images of serial sections was used to independently classify fibers as Type I (labelled by A4.951 only) or Type II (labelled by A4.74 only). These classifications were then compared to determine hybrid (labelled by both A4.951 and A4.74) and unlabelled (labelled by neither A4.951 nor A4.74) fibers. Classification was performed for at least one thousand fibers in total from each sample, and fiber classification for all eleven specimens was performed by the same investigator (KS). Data were entered into a Microsoft Excel spreadsheet, where the percentages of Type I, Type II, hybrid and unlabelled fibers in total classified fiber counts were determined for each specimen.

### Statistical analyses

The statistical package SPSS Version 18.0 (SPSS Inc., Chicago, USA) was utilized for all statistical analyses. Descriptive statistics were used to evaluate the mean percentage, standard deviation, and range of values obtained through fiber type calculations. Paired samples t-tests were performed to compare mean percentages of fiber types and muscle heads. In all statistical evaluations, the level of significance was set at *P < 0.05*.

## Results

**Figure 3:**
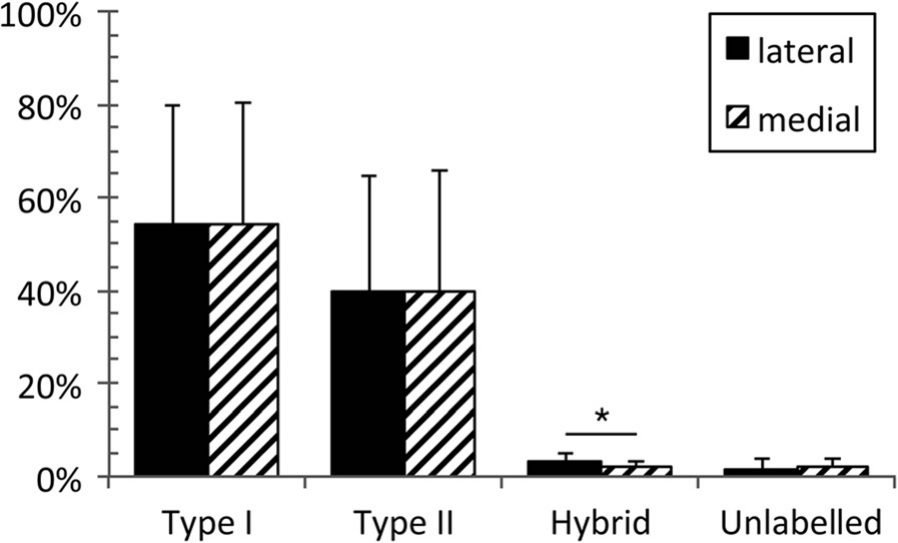
**Comparison of mean percentage of fiber types between lateral and medial heads of the quadratus plantae.** Values represent sample population means, and error bars represent standard deviation. A statistically significant difference (*P < 0.05*) was found only in hybrid fiber content between lateral and medial heads, denoted by an asterisk. Results show mean percentages of Type II and Type II fibers are not significantly different between lateral and medial heads.

**Figure 4:**
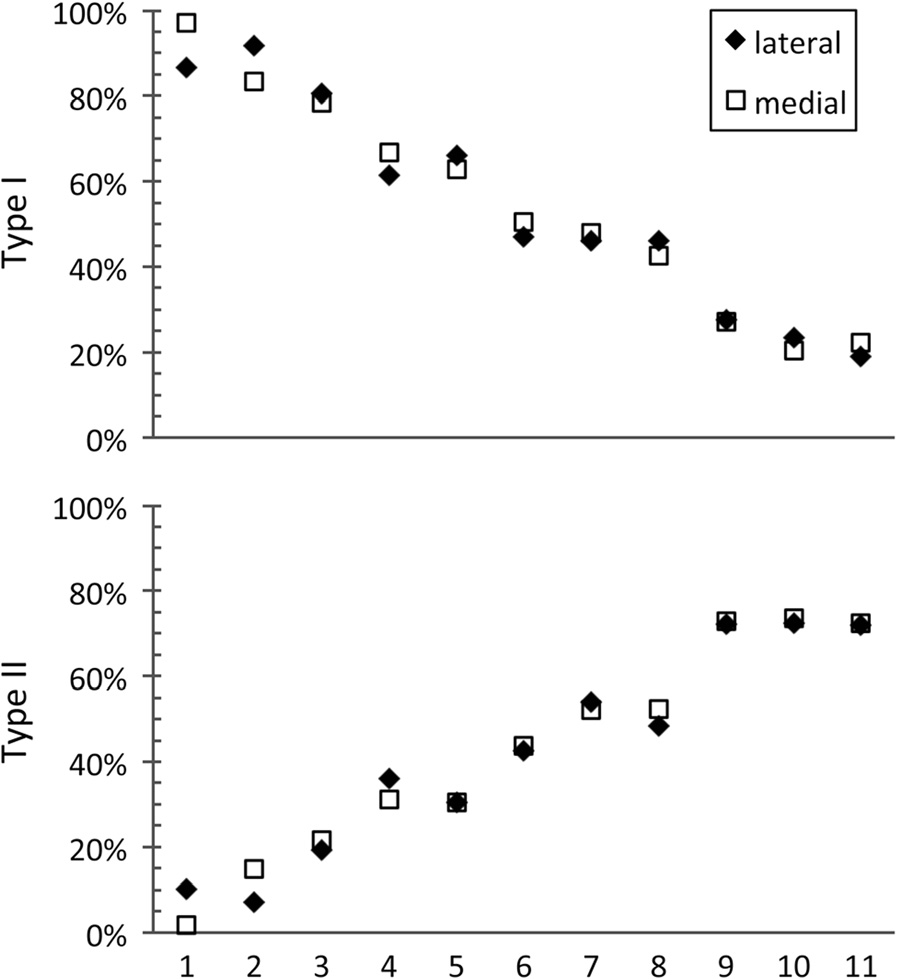
**Comparison of Type I and Type II fiber content between lateral and medial heads of individual quadratus plantae.** Values represent the fiber type percentages of individual specimens, arranged from highest to lowest mean Type I fiber percentage. Results demonstrate that fiber type composition is very similar within lateral and medial heads of an individual, but that overall fiber type composition varies widely across the sample population.

Mean percentages of fiber types within the lateral and medial heads of the human quadratus plantae muscle are presented in Figure 3. Both medial and lateral heads were composed predominantly of Type I and Type II fibers, with no significant difference in fiber type prevalence between these regions (Type I, *P = 0.85,* 95% CI [-3.8, 3.2]; Type II, *P = 0.86*, 95% CI [-3.0, 2.6]). There was no significant difference in the mean percentages of Type I and Type II fibers within each head (lateral, *P = 0.44*, 95% CI [-21.2, 45.1]; medial, *P = 0.45*, 95% CI [-21.8, 45.8]). A high degree of variation was observed in individual percentages of Type I and II fibers (Figure 4). Type I fiber percentages ranged from 19.1% to 91.6% within the lateral head (mean, 54.2%), and from 20.4% to 97.0% within the medial head (mean, 54.5%). The percentage of Type II fibers ranged from 7.1% to 72.5% (mean, 39.9%) and from 1.8% to 73.6% (mean, 40.0%) within lateral and medial heads, respectively. The average percentage point difference between lateral and medial heads of an individual was 4.1% (range, 0.6% to 10.4%) and 2.9% (range, 0.0% to 8.3%) for Type I and Type II fibers (Figure 4). In general, both Type I and Type II fibers were observed to be unevenly distributed throughout the tissue, forming clusters or regions of predominantly one fiber type (Figure 2). Fiber sizes could not be quantified, as dehydration-related fiber shrinkage precluded accurate measurement of this metric.

Hybrid fibers were present as a minor component of total fiber composition in both lateral and medial quadratus plantae, with the lateral head having significantly more hybrid fibers than the medial head (*P = 0.03,* 95% CI [0.2, 1.9]). The mean percentage of hybrid fibers within the lateral head was 3.3% (range 1.2% to 5.5%), and within the medial head 2.3% (range 0.9% to 4.2%).

A small number of structures were present that morphologically resembled muscle fibers but did not label with antibodies against Type I or II myosin. These unlabelled structures were confirmed to be muscle fibers through the detection of myosin heavy chains by antibody A4.1025 (not shown). The mean percentage of unlabelled fibers was not statistically different between lateral and medial heads (*P = 0.69,* 95% CI [-2.1, 1.5]). In all specimens the percentage of fibers classified as unlabelled was below 5.5%, with an average percentage of 1.7% and 2.0% for lateral and medial heads, respectively.

## Discussion

To our knowledge this is the first study to investigate the fiber type composition of the quadratus plantae muscle. The principal findings of this study are (1) the ratio of fiber types within the quadratus plantae is highly variable among older adults and (2) the fiber type content of the medial and lateral heads of the quadratus plantae is highly similar within an individual. The finding that the two heads of the quadratus plantae are similar in fiber type composition supports the lateral and medial heads as having a singular function. As description of the quadratus plantae has come primarily from dissection studies and magnetic resonance imaging, information about the fiber type composition will contribute to better understanding the role of this muscle.

It has been theorized that functionally distinct neuromuscular compartments may arise from parent muscle during evolution, forming separate regions or even new muscles [25-27]. This process is thought to have occurred at the distal end of flexor hallucis longus, giving rise to the medial head of quadratus plantae in humans [6,10,11,28,29]. Support for this theory comes primarily from the existence of flexor digitorum accessorius longus, an anomalous muscle present in 6-12% of the human population that is thought to represent an intermediate in part of flexor hallucis longus descending into the sole [6,30-32]. The evolutionary appearance of the medial quadratus plantae in humans may be related to the demands of bipedalism, as first suggested by Wood Jones [28]. The medial quadratus plantae may assist in maintaining foot eversion in a bipedal stance [7,11], or in stabilizing the flexed toes against the pull of the leg extensors during locomotion [5,28]. Our finding of fiber type homogeneity between the lateral and medial quadratus plantae suggests both heads have a shared function, though the origins are from discrete parts of the calcaneus. This implies that the development of the medial quadratus plantae in humans may not be tied to adding additional function to the muscle, but perhaps increasing muscle bulk or strengthening attachment points to support increased postural demand on the lower limb.

Mature human muscles are composed primarily of Type I and Type II fibers [14,15], with fiber types being classified based on the predominantly expressed myosin heavy chain isoform [17]. Expression of multiple myosin heavy chain isoforms can result in the appearance of hybrid fibers [33], which may represent a typical fiber phenotype, fibers undergoing inter-type transformation during reinnervation, or regenerating fibers [15-18]. Our study detected a low percentage of hybrid fibers in all studied specimens, which is similar to what has previously been reported for Type I/II hybrid fibers in the vastus lateralis muscle in the elderly [34]. We detected significantly more hybrid fibers within the lateral head, which may reflect differential wear, however as the proportion of hybrid fibers was small it is difficult to interpret the functional implications of this difference. In addition to the Type I, Type II, and hybrid fibers described in our study, structures were detected that contained myosin isoforms not labelled by Type I- or Type II-specific antibodies. These unlabelled fibers may contain immature embryonic and/or developmental myosin, which can be re-expressed in aging muscle fibers undergoing regeneration or atrophy [15,35]. Alternatively, these fibers may represent fibers containing solely the Type IIX myosin isoform [23,24].

The effects of aging on muscle tissues have been well described, and include phenomena such as an overall reduction in fiber number and changes in fiber morphology [36,37]. In our current study we observed a wide range of fiber type compositions in the quadratus plantae muscle, which may represent normal variation due to genetic or physiological differences, degenerative processes, or some combination thereof. Differences in genetic makeup are estimated to account for 40-50% of the Type I fiber proportion variance observed in a population, with the remainder attributable to environmental factors such as nutrition and activity levels [13,38]. Physiological differences in foot architecture could contribute to variation in quadratus plantae fiber type composition, as differences in load distribution by the foot may affect recruitment of this muscle and in turn its fiber type composition [39-41]. Injuries to the foot, reduced range of motion, and even altered sensory perception of the plantar surface could also affect loading patterns of the foot during gait [42-44], potentially changing demand on the quadratus plantae. Information regarding mobility status and activity history was not available for our present study, which is common of most cadaveric studies, and it was not therefore possible to assess the effects of these functional or architectural variables in our population.

Degenerative processes may also have contributed to the range of fiber type compositions observed. Atrophy following muscle disuse is accompanied by slow-to-fast fiber type transitions [16,45,46], which may be involved in a muscle becoming composed of predominantly Type II fibers. Similarly, reinnervation in aging muscle can feature the preferential loss of Type II fibers [36,47], which may have affected individuals with a predominantly Type I composition. Within the quadratus plantae fiber type grouping, or the appearance of muscle fibers in patches of predominantly one fiber type, was commonly observed in all our studied specimens. Few areas of quadratus plantae tissue examined in this study exhibited the checkerboard pattern of Type I and Type II fibers typical of healthy muscle. Fiber type grouping has been observed in studies with a similar sample population age [48], and is consistent with the cumulative effects of muscle denervation and reinnervation associated with aging muscle [15,49].

The difficulties inherent to obtaining young cadaveric specimens are well known. As only a limited number of specimens were available for research use at our institution, a large number of male and female specimens were not obtainable. As such, the relationship between variables such as age or sex and the fiber type composition of the quadratus plantae were not able to be assessed within our sample. Despite these limitations, our data showed remarkable homogeneity between the two heads of the quadratus plantae throughout a highly variable population. As a degree of inaccuracy is inherent to measuring the fiber type composition of a muscle [50], the finding of homogeneity over a wide range of compositions indicate our results and conclusions to be robust. Future work to examine the quadratus plantae fiber type composition in a younger cohort would be useful in delineating the effects of advancing age on the muscles of the foot.

## Conclusions

In conclusion, the results of this study are the first to depict the spectrum of fiber type composition in the quadratus plantae of normal older adults. Our finding that the lateral and medial quadratus plantae have highly similar fiber type composition within an individual supports this muscle having a singular function. Our results also demonstrated a high degree of variation in the fiber type ratios, which may reflect pathological or normal physiological states.
